# Drinking Non-nutritive Sweetness Solution of Sodium Saccharin or Rebaudioside a for Guinea Pigs: Influence on Histologic Change and Expression of Sweet Taste Receptors in Testis and Epididymis

**DOI:** 10.3389/fnut.2021.720889

**Published:** 2021-08-05

**Authors:** Ting Shen, Junrong Li

**Affiliations:** ^1^College of Agriculture, Jinhua Polytechnic, Jinhua, China; ^2^College of Animal Science, Zhejiang University, Hangzhou, China

**Keywords:** sodium saccharin, rebaudioside A, guinea pigs, sweet taste receptor, testis, epididymis, non-nutritive sweetness

## Abstract

Saccharin sodium and rebaudioside A are extensively used as non-nutritive sweeteners (NNSs) in daily life. NNSs elicit a multitude of endocrine influences on animals, differing across species and chemically distinct sweeteners, whose exposure induce activation of sweet taste receptors in oral and extra-oral tissues with consequences of metabolic changes. To evaluate the influence of NNSs on histologic change and expression of sweet taste receptors in testis and epididymis of young male guinea pigs, thirty 4-week-old male guinea pigs with body weight 245.73 ± 6.02 g were randomly divided into five groups (*n* = 6) and received normal water (control group) and equivalent sweetness low dose or high dose of sodium saccharin (L-SS, 1.5 mM or H-SS, 7.5 mM) or rebaudioside A (L-RA, 0.5 mM or H-RA, 2.5 mM) solution for 28 consecutive days. The results showed that the relative testis weight in male guinea pig with age of 56 days represented no significant difference among all groups; in spite of heavier body weight in L-SS and H-RA, NNS contributes no significant influence on serum testosterone and estradiol level. Low-dose 0.5 mM rebaudioside A enhanced testicular and epididymal functions by elevating the expressions of taste receptor 1 subunit 2 (T1R2) and gustducin α-subunit (GNAT3), and high-dose 7.5 mM sodium saccharin exerted adverse morphologic influences on testis and epididymis with no effect on the expression of T1R2, taste receptor 1 subunit 2 (T1R3), and GNAT3. In conclusion, these findings suggest that a high dose of sodium saccharin has potential adverse biologic effects on the testes and epididymis, while rebaudioside A is a potential steroidogenic sweetener for enhancing reproductive functions.

## Introduction

Taste or gustation is one of the five traditional senses including hearing, sight, touch, and smell, and the sense of taste has classically been limited to the five basic taste qualities: sweet, salty, sour, bitter, and umami or savory (1). Taste sense is crucial for many organisms. For example, taste has a nutrient-signaling function, which in turn affects the sensory stimulation to eat and consume (2). Liking for sweet-tasting substance is innate. The percentage ratio of sweet taste to bitter taste may be a better gauge of the broadly conceived food value of a plant than sweetness alone (3). Sweet and bitter and taste-receptor cells provide instructive signals to sweet and bitter target neurons *via* different guidance molecules (4). Most sweet compounds show lower hydrophobicity compared with bitter compounds, while sweet molecules have a wider range of sizes, and further focus on polypharmacology may unravel new physiological roles for tastant molecules (5). Taste signal-modifying factors, such as serum components, may have a contributing role in aging-related changes in taste sensitivity (6). Taste perception has been investigated in “taste” and “no-taste” tissues, and components of taste transduction cascade in the testis are found to be involved in spermatogenesis (7), but functional implications of taste senses in the field of male reproduction remain unclear despite recent advances.

Non-nutritive sweeteners (NNSs), known as artificial sweeteners, high-intensity sweeteners, or non-caloric sweeteners are ubiquitously used as sugar substitutes in soft drinks, processed grains, and dairy products. The data on NNSs and body weight are inconsistent and not beneficial for weight loss, and overweight or obese individuals are more likely to consume low-calorie foods and beverages; whether NNSs have a relationship with abnormal changes in gut microbiota requires further study (8). In particular, certain artificial sweetener effects on the body remain not incontrovertible, for example, sucralose has a physiological influence of changing glucose metabolism (9). Different to the testicular degeneration in rat exposed to high-dose nutritive sweetener fructose (10) and the adverse effects of high daily intake of sugar on pregnant rat and offspring (11), the low-dose natural NNS stevia rebaudianabertoni extract increased ejaculation frequency and intromission frequency with aphrodisiac properties in streptozotocin-induced diabetic male rats (12). Meanwhile, there was no observed adverse effect of D-allulose treatment on gender ratios, viability indexes, and prenatal death rate in rats (13). NNSs elicit a multitude of endocrine influences *in vivo*, in animal models and in humans differing across species and chemically distinct sweeteners, whose exposure induces activation of sweet taste receptors in oral and extra-oral tissues with consequences of metabolic changes (14).

Sweet taste receptor is a heterodimer of class C G-protein coupled receptors comprising taste receptor 1 subunit 2 (T1R2) and taste receptor 1 subunit 3 (T1R3. However, the structure of the sweet taste receptor is still undetermined (15). It was proposed that identified taste receptors and coupled signaling cascades keep sperm in a chronically quiescent state until they arrive in the vicinity of the egg, either by constitutive receptor activity and/or by tonic receptor activation (16). Rodent investigation results indicated a crucial role of extra-oral sweet taste receptor in sperm development and maturation, clofibrate inhibition of humanized T1R3 in the genetic background of T1R3(–/–), gustducin α-subunit GNAT3(–/–) doubly null mice led to inducible male sterility, T1R3 and GNAT3 activators were speculated to help infertile men (17). Sweet taste receptor is widely expressed in testis, where T1R3 and heterotrimeric G protein α-gustducin (Gα) exhibit a stage-dependent expression pattern during testicular development and a cell-specific pattern during the spermatogenic cycle in male mice (18). A previous study has revealed that 35 days of exposure to high-dose 7 mM saccharin deduced weaker immunoreactions of anti-T1R3 antibodies on Leydig cells and VII-VIII stages of spermatids, and saccharin-induced physiologic effects on mouse testis are associated with testicular T1R3 and Gα, which differed from sucrose (19). However, the testicular and epididymal function of different sweeteners remains uncertain, and the biological features of sweet taste receptor in testis and epididymis are still poorly understood. This study was designed to investigate the testicular and epididymal influence of two types of non-nutritive sweeteners on body weight, reproductive hormone, and morphological change, associating with the expressions of T1R2, T1R3, and GNAT3 in testis and epididymis of reproductive animal model guinea pigs.

## Materials and Methods

### Experimental Design and Ethics Statement

Thirty 4-week-old male Harley-white guinea pigs from Zhejiang Chinese Medical University with body weight 245.73 ± 6.02 g were randomly divided into five groups (*n* = 6) and received drinking normal water (control group) and equivalent sweetness low dose or high dose of sodium saccharin (1.5 mM or 7.5 mM) or rebaudioside A (0.5 or 2.5 mM) solution for 28 consecutive days. Food and drinking water were provided *ad libitum*. The animals were housed (room temperature: 22 ± 1°C, relative humidity of 30–40%, light with 150-200 Lx from 8 am to 8 pm, and noise: below 50 dB) in the Zhejiang Chinese Medical University Laboratory Animal Research Center with experiment facility license of SYXK(ZHE) 2018-0012, and the animal experiment process conforms to the principle of animal protection, animal welfare and ethics, and related stipulation on the National Experimental Animal Welfare Ethics (China) with approval number of IACUC-20181224-13.

### Food Intake, Water Consumption, and Weight of Body, Testis, and Epididymis

Food intake and water consumption were measured every day at 9 am and represented by weekly average data in figures, group average daily data = overall feed intake or water consumption of two cages (three animals per cage) in same group/number of animals in the group. The change in body weight was analyzed by weight determination on days 1, 7, 14, 21, and 28; weights of testis and epididymis on the right side were measured on day 28.

### Sample Collection and Determination of Serum Testosterone and Estradiol

The animals were euthanized by CO_2_ anesthesia with the guidelines of the Care and Use of Laboratory Animals prepared by the Institutional Animal Care and Use Committee of Zhejiang Chinese Medical University. Blood samples were immediately collected by cardiac puncture and centrifuged for serum (5,000 rpm, 10 min). The serum concentration of testosterone and estradiol was determined with a commercial enzyme-linked immunosorbent assay (ELISA) kit (Nanjing Jiancheng Bioengineering Institute, Jiangsu, China). Testis and epididymis samples from the right side were weighted and fixed in 4% paraformaldehyde for H&E and immunohistochemistry (IHC), and samples from the left side were stored at −80°C for Western blotting (WB) analysis.

### Hematoxylin-Eosin Staining and Immunohistochemistry Observation

The fixed samples were embedded in paraffin and serially sectioned at 4 μm. H&E tissues were stained with H&E (Nanjing Jiancheng Bioengineering Institute, Jiangsu, China). IHC tissues were heated in 10 mM sodium citrate buffer for 8 min in a microwave oven at 100°C. Endogenous peroxidase activity and non-specific binding were blocked with 0.3% H_2_O_2_ in phosphate-buffered saline (PBS) for 30 min. Slides were incubated overnight with primary antibodies (diluted at 1:100 in PBS, Abcam, Cambridge, United Kingdom) to T1R2 (ab150495, lot: GR3198736-7), T1R3 (ab150525, lot: GR155874-26), and GNAT3 (ab107512, lot: GR37772-10) at 4°C, detected with anti-rabbit IgG (PV-8000, lot: 191030326, 1:1 ready to use, ZSGB-BIO, China) for 60 min and finally visualized with diaminobenzidine (DAB, Sigma-Aldrich, St. Louis, MI, United States) as substrate and counter-stained with hematoxylin. The negative control tissues were incubated with normal rabbit serum (NRS) instead of primary antibodies. All stained sections were scanned with a light microscope Nikon Eclipse 80i (Nikon, Tokyo, Japan) and analyzed on the viewer software NDP (Hamamatsu, Japan). Protein immunolocation of T1R2, T1R3, and GNAT3 was analyzed by positive immunostaining in brown and counterstained with hematoxylin.

### Western Blotting Determination

The −80°C-stored samples were homogenized in radio-immunoprecipitation assay (RIPA) buffer with 10 mM phenylmethylsulfonyl fluoride (PMSF). Equal amounts of protein lysate (50 μg) were separated by 10% (w/v) sodium dodecyl sulfate–polyacrylamide gel electrophoresis (SDS-PAGE) (Sangon Biotech, Shanghai, China) and electro-transferred onto polyvinylidene difluoride (PVDF) membranes (Millipore, Burlington, MA, United States). Membranes were blocked with 3% bovine serum albumin (BSA) (BBI, Shanghai, China) for 2 h at 25°C and incubated with primary antibodies (diluted at 1:500 in PBS) of T1R2, T1R3, GNAT3 (same as IHC antibodies, Abcam, Cambridge, United Kingdom), and glyceraldehyde 3-phosphate dehydrogenase (GAPDH) (lot: HG0718, HuaBio, Hangzhou, China) for 15 h at 4°C. The incubated membranes were washed with Tris-buffered saline with Tween (TBST) buffer and second-incubated with NIR-secondary antibody Odyssey IRDye 680RD goat anti-rabbit IgG (lot: C51104-08, LI-COR, Lincoln, NE, United States) for 1.5 h at 25°C, respectively. The bands were washed in TBST buffer (five times) and visualized with an Odyssey CLx imaging system (LI-COR, Lincoln, NE, United States), and the intensities of blots were calculated with target protein bands to corresponding GAPDH.

### Statistical Analysis

All numerical results are expressed as mean ± SD and are analyzed with Prism 8 version 8.0.2 (GraphPad Software, San Diego, CA, United States). One-way ANOVA was performed for analyzing testicular weight, serum hormone concentration, and WB intensities; daily food intake, water consumption, and body weight were evaluated by two-way ANONA. Tukey's range test was applied for multiple comparisons, and *p* < 0.05 was considered to be significant.

## Result

### Food Intake and Water Consumption

**Figure 1:**
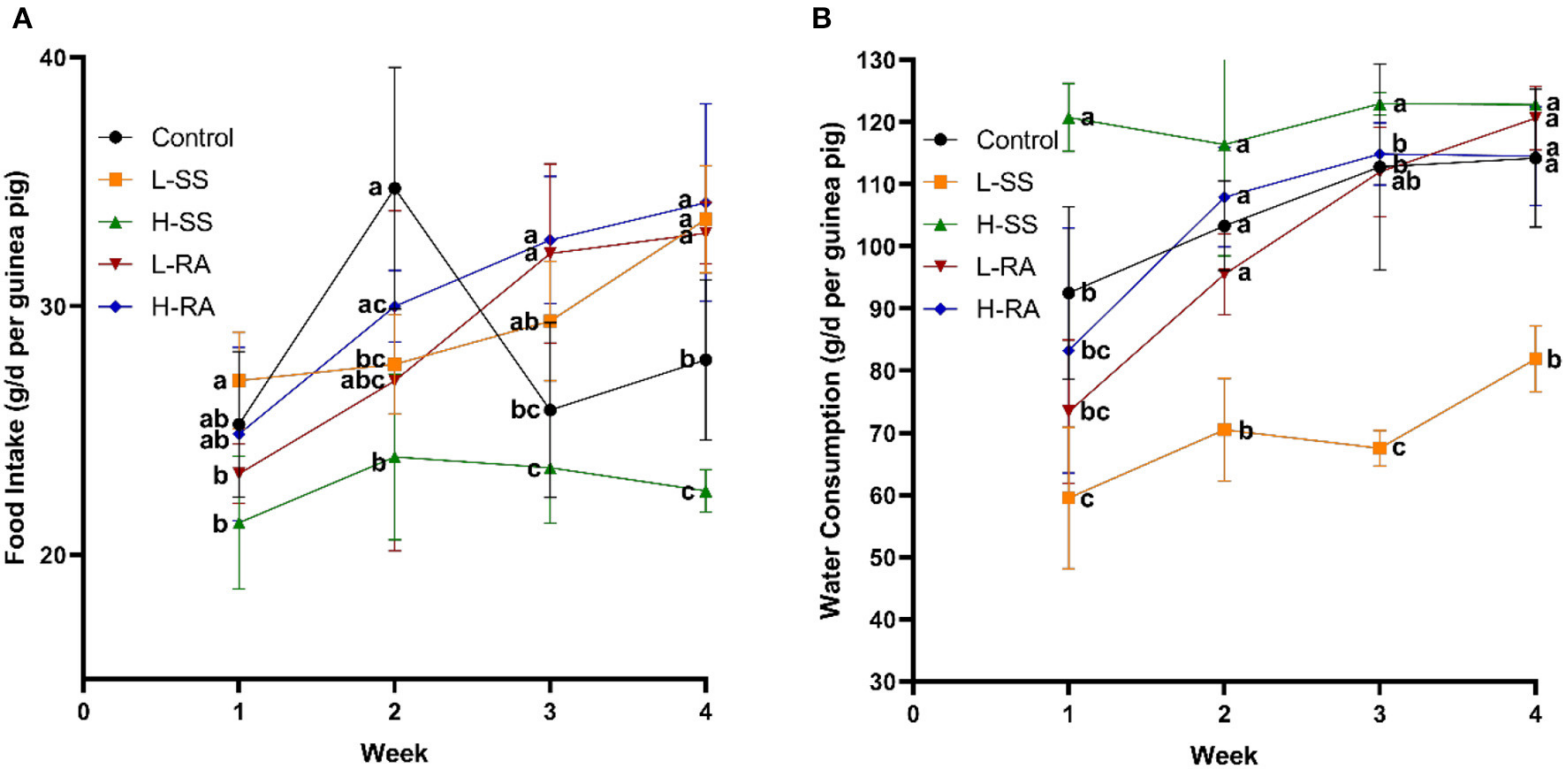
Effect of dietary non-nutritive sweetness drinking on food intake and water consumption in male guinea pigs. Animals in the control, L-SS, H-SS, L-RA, and H-RA groups were supplied water solution with normal water, 1.5 mM sodium saccharin, 7.5 mM sodium saccharin, 0.5 mM rebaudioside A, and 2.5 mM rebaudioside A for 28 days, respectively. Each point in figures represents average daily food intake **(A)** and water consumption **(B)** per week. Data are shown as means ± SD (*n* = 6). Points in the same vertical line with different letters denote significant differences (*P* < 0.05).

Non-nutritive sweetness drinking affected both food intake (Figure 1A) and water consumption (Figure 1B) of the male guinea pigs from weeks 1 to 4. Compared with the control group, low-dose sodium saccharin significantly reduced water consumption from weeks 1 to 4 (Figure 1B, *P* < 0.05), high-dose sodium saccharin significantly reduced food intake in weeks 2 and 4, and low-dose and high-dose rebaudioside A both significantly elevated food intake in weeks 3 and 4 (Figure 1A, *P* < 0.05). Interestingly, rebaudioside A represented no significant difference in food intake and water consumption with low-dose or high-dose. However, high-dose sodium saccharin contributed to significantly lower food intake and higher water consumption in weeks 1, 3, and 4 compared with low-dose sodium saccharin (*P* < 0.05).

### Body Weight, Testis Weight, and Epididymis Weight

**Figure 2:**
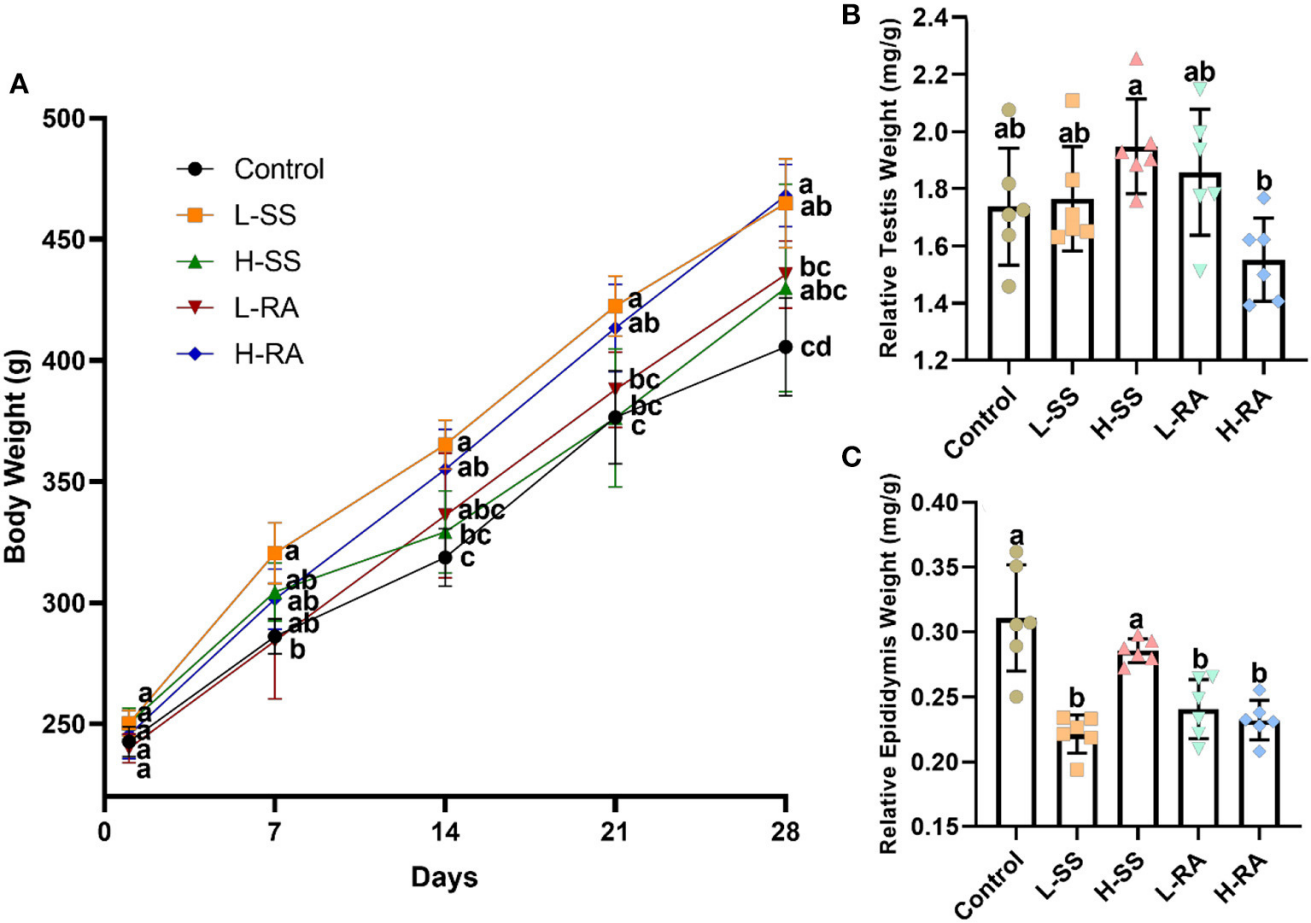
Effect of dietary non-nutritive sweetness drinking on body weight, relative testis weight, and epididymis weight (testis weight or epididymis weight/body weight) in male guinea pigs. Animals in control, L-SS, H-SS, L-RA and H-RA groups were supplied water solution with normal water, 1.5 mM sodium saccharin, 7.5 mM sodium saccharin, 0.5 mM rebaudioside A, and 2.5 mM rebaudioside A for 28 days, respectively. Each point in **(A)** represents average body weight at days 1, 7, 14, 21, and 28, testis **(B)**, and epididymis **(C)** weights were measured at day 28. Data are shown as means ± SD (*n* = 6). Points in the same vertical line on **(A)** with different letters denote significant differences (*P* < 0.05), bars shown on **(B,C)** with different letters denote significant differences (*P* < 0.05).

There was no significant difference in body weight among all the groups on day 1, while there was no significant difference in body weight during all the experimental periods among control, H-SS, and L-RA (Figure 2A, *P* > 0.05). However, non-nutritive sweetness drinking increased the body weight on day 21 and day 28 in L-SS and H-RA compared with that in control (Figure 2A, *P* < 0.05). Compared with control, male guinea pigs exposed to sodium saccharin or rebaudioside A manifested no significant changes in the relative testis weight in all the treated groups (Figure 2B, *P* > 0.05). However, the relative epididymis weight in groups L-SS, L-RA, and H-RA was significantly lower than that in the control group (Figure 2C, *P* < 0.05).

### Serum Concentration of Testosterone and Estradiol

**Figure 3:**
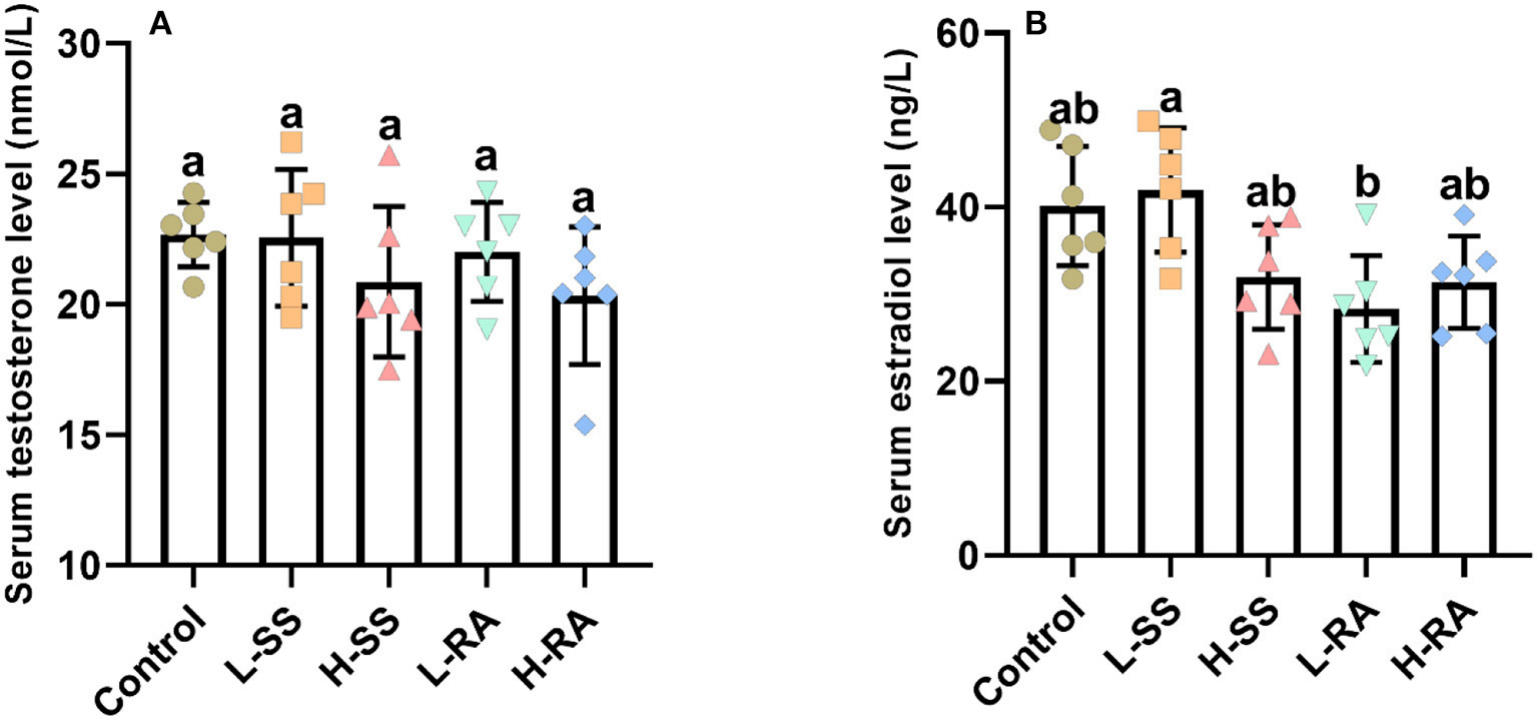
Effect of dietary non-nutritive sweetness drinking on serum concentration of testosterone and estradiol in male guinea pigs. Animals in the control, L-SS, H-SS, L-RA, and H-RA groups were supplied water solution with normal water, 1.5 mM sodium saccharin, 7.5 mM sodium saccharin, 0.5 mM rebaudioside A, and 2.5 mM rebaudioside A for 28 days, respectively. Blood samples were collected and centrifuged for the serum to measure testosterone **(A)** and estradiol **(B)** concentration by ELISA. Data are shown as means ± SD (*n* = 6). Bars with different letters denote significant differences (*P* < 0.05).

The serum concentration of testosterone was not significantly different in all groups (Figure 3A, *P* > 0.05), drinking of low-dose rebaudioside A solution reduced the serum concentration of estradiol, which was significantly lower in L-RA than that in L-SS (Figure 3B, *P* < 0.05).

### Morphologic Observations of Testis and Epididymis

**Figure 4:**
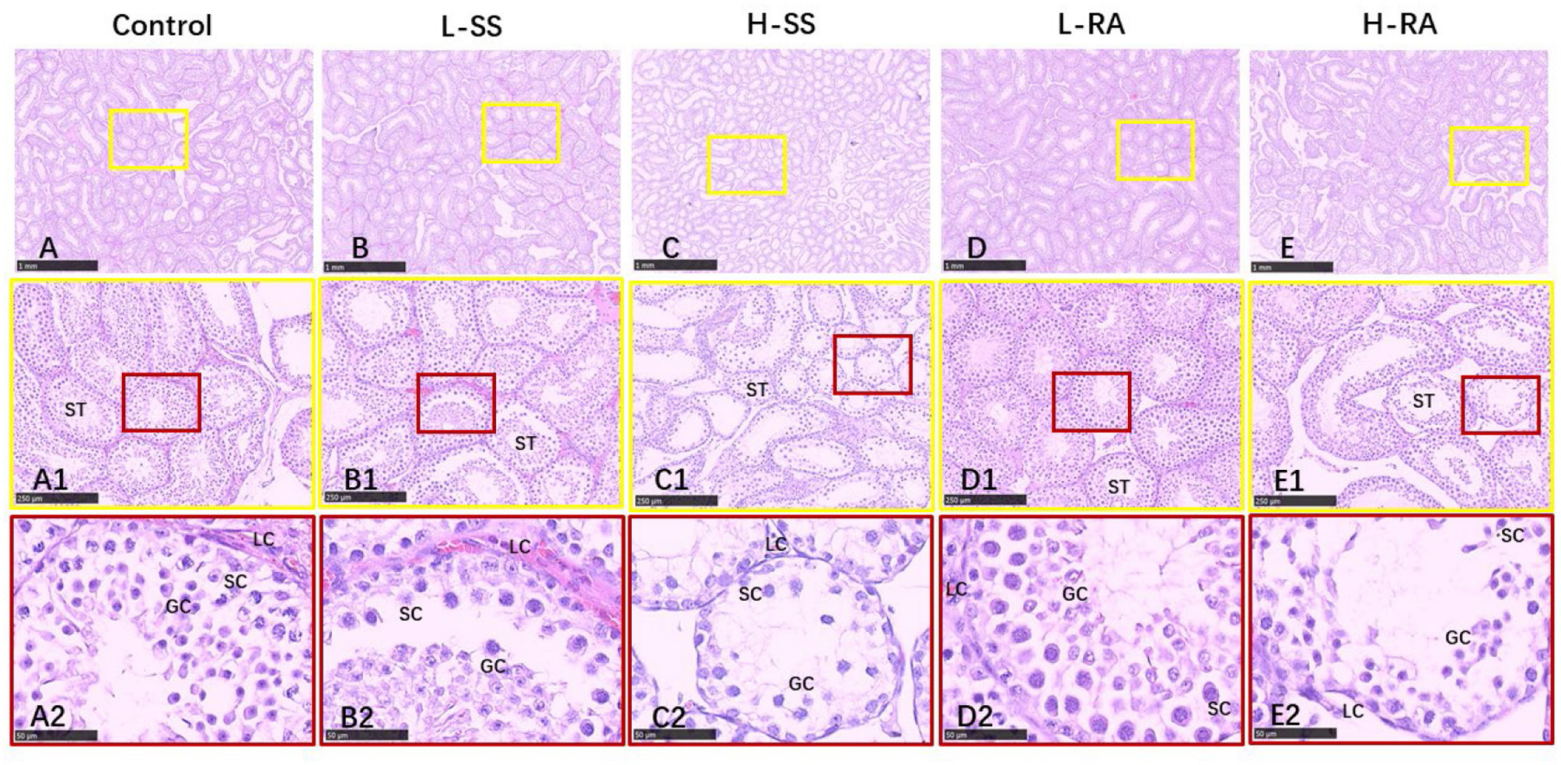
Effect of dietary non-nutritive sweetness drinking on histologic changes in the testis of male guinea pigs. Animals in the control, L-SS, H-SS, L-RA, and H-RA groups were supplied water solution with normal water, 1.5 mM sodium saccharin, 7.5 mM sodium saccharin, 0.5 mM rebaudioside A, and 2.5 mM rebaudioside A for 28 days, respectively. Tissues were sectioned at 4 μm and stained with H&E. Graphs in small colored square boxes are enlarged below. GC, germ cell; SC, Sertoli cell; LC, Leydig cell. Bars = 1,000 μm **(A–E)**, 250 μm **(A1–E1)**, and 50 μm **(A2–E2)**.

We found that seminiferous tubules in control were regularly connected with Leydig cells in testis (Figure 4A1), containing a large number of germ cells (spermatogonia, spermatocyte, and spermatid) and Sertoli cells (Figure 4A2). Compared with control, non-nutritive sweetness drinking caused certain damage to the periphery and lumen inside of seminiferous tubules (Figures 4A–E, 4B1–E1). Obviously, high-dose sodium saccharin markedly reduced the number of Leydig cells adjacent to seminiferous tubules, with rare germ cells and Sertoli cells (Figure 4C2). Meanwhile, germ cells were exfoliated from the Sertoli cells in L-SS and H-RA (Figures 4B2,E2), and cohesive germ cells with different developing stage were observed in L-RA (Figure 4D2).

**Figure 5:**
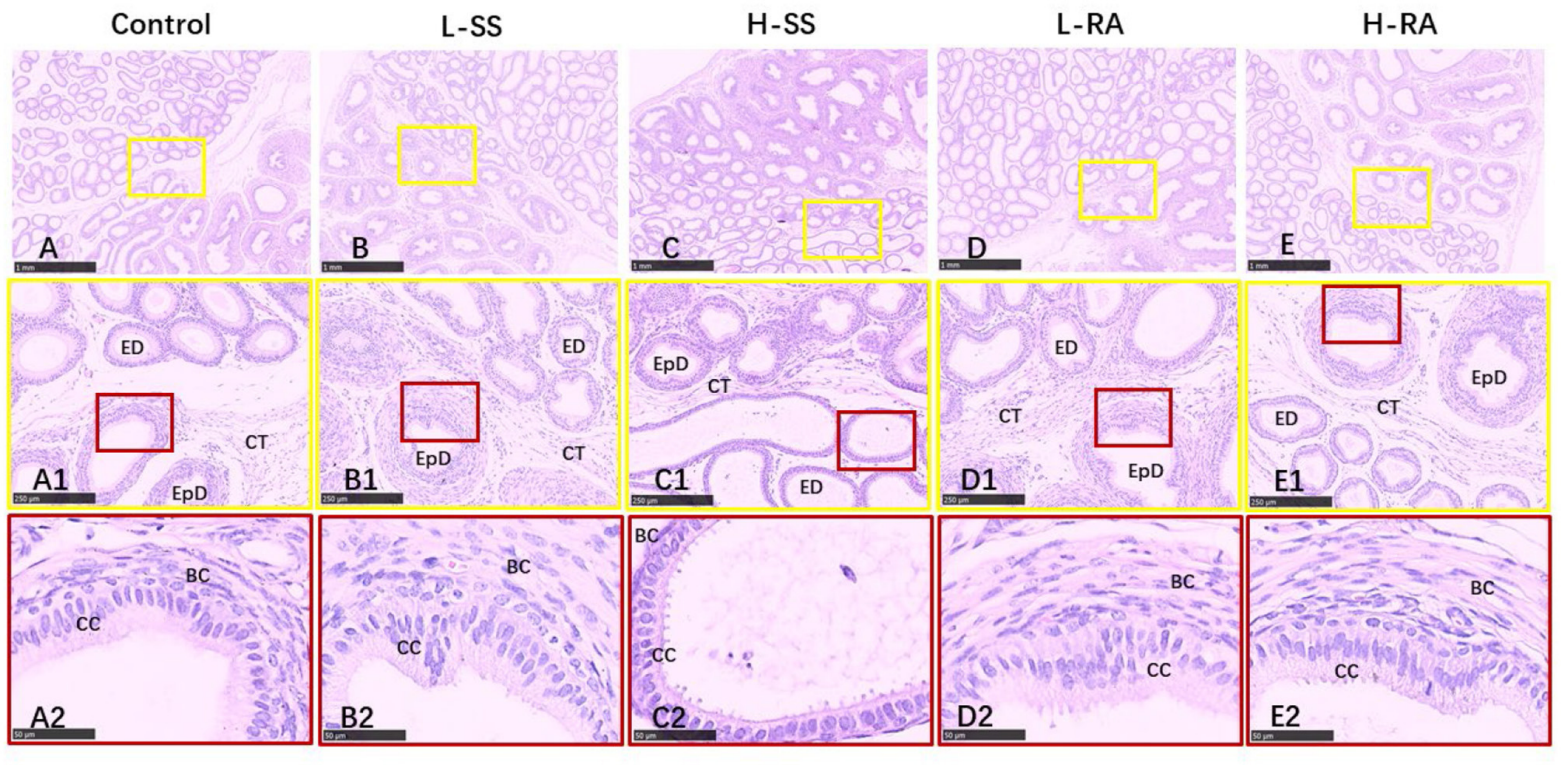
Effect of dietary non-nutritive sweetness drinking on histologic changes in the epididymis in male guinea pigs. Animals in the control, L-SS, H-SS, L-RA, and H-RA groups were supplied water solution with normal water, 1.5 mM sodium saccharin, 7.5 mM sodium saccharin, 0.5 mM rebaudioside A, and 2.5 mM rebaudioside A for 28 days, respectively. Tissues were sectioned at 4 μm and stained with H&E. Graphs in small colored square boxes are enlarged below. ED, efferent duct; EpD, epididymis ductus, CT, connective tissue; CC, ciliated cell; BC, basal cell. Bars = 1,000 μm **(A–E)**, 250 μm **(A1–E1)**, and 50 μm **(A2–E2)**.

In order to assess the influence of non-nutritive sweetness exposure on sperms or late spermatids outside of testis, we also examined morphologic changes in paraffin-embedded tissues of the epididymis (Figures 5A–E). Unlike the obvious damages in testis, there appeared to be no serious damage in the efferent duct, connective tissues, and epididymis ductus (Figures 5A1–E1), with a visible thick layer of basal cells in epididymis ductus compared with control (Figures 5A2,B2,D2,E2). Exceptionally, the elongated and enlarged efferent ducts with a single layer of ciliated cells were observed in H-SS (Figures 5C1,C2).

### Immunohistochemistry Examination of Testis and Epididymis

**Figure 6:**
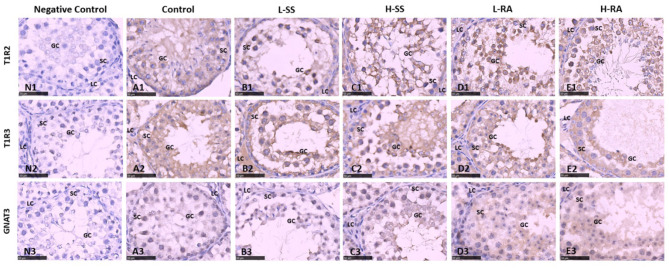
Effect of dietary non-nutritive sweetness drinking on immunolocalization of the sweet-tasting molecules T1R2 **(6A1–E1)**, T1R3 **(6A2–E2)**, and GNAT3 **(6A3–E3)** in the seminiferous tubule of testis in male guinea pigs. Animals in the control, L-SS, H-SS, L-RA, and H-RA groups were supplied water solution with normal water, 1.5 mM sodium saccharin, 7.5 mM sodium saccharin, 0.5 mM rebaudioside A, and 2.5 mM rebaudioside A for 28 days, respectively. Tissues were sectioned at 4 μm and immunostained with primary antibody to T1R2, T1R3, and GNAT3 proteins, and negative control was performed with NRS instead of primary antibody **(6N1–N3)**. Positive immunostaining in brown and counterstaining with hematoxylin. GC, germ cell; SC, Sertoli cell; LC, Leydig cell. Bars = 50 μm.

The sweet taste receptors T1R2, T1R3, and GNAT3 were positively immunostained in germ cells, Sertoli cells, and Leydig cells in the seminiferous tubule of testis in the male guinea pigs (Figure 6). We found that the immunoreactions of anti-T1R2 and anti-GNAT3 antibodies on germ cells and Sertoli cells in L-RA and H-RA were stronger than those in control, L-SS, and H-SS (Figures 6A1–E1,A3–E3). However, the anti-T1R3 antibodies showed no specific difference in immunoreactions on germ cells and Sertoli cells among all the groups (Figures 6A2–E2).

**Figure 7:**
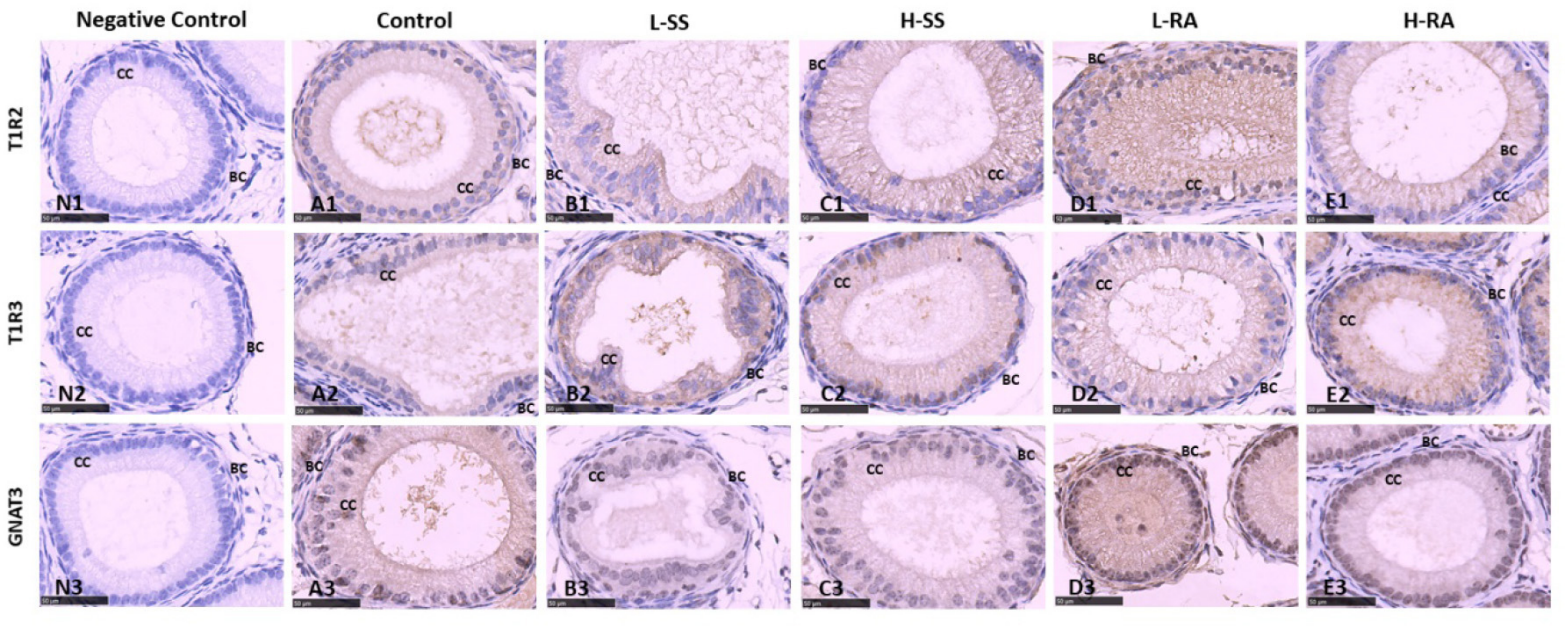
Effect of dietary non-nutritive sweetness drinking on immunolocalization of the sweet-tasting molecules T1R2 **(7A1–E1)**, T1R3 **(7A2–E2)**, and GNAT3 **(7A3–E3)** in the efferent duct of the epididymis in male guinea pigs. Animals in the control, L-SS, H-SS, L-RA, and H-RA groups were supplied water solution with normal water, 1.5 mM sodium saccharin, 7.5 mM sodium saccharin, 0.5 mM rebaudioside A, and 2.5 mM rebaudioside A for 28 days, respectively. Tissues were sectioned at 4 μm and immunostained with primary antibody to T1R2, T1R3, and GNAT3 proteins, and negative control was performed with NRS instead of primary antibody **(7N1–N3)**. Positive immunostaining in brown and counterstaining with hematoxylin. CC, ciliated cell; BC, basal cell. Bars = 50 μm.

In guinea pig epididymis, the sweet taste receptors T1R2, T1R3, and GNAT3 were positively immunostained in basal cells and ciliated cells (Figure 7). Similar to the testicular immunoreactions, we also found that the immunoreactions of anti-T1R2 and anti-GNAT3 antibodies on basal cells and ciliated cells in L-RA were stronger than those in control, L-SS, and H-SS (Figures 7A1–E1,A3–E3). The anti-T1R3 antibodies showed no specific difference in immunoreactions on basal cells and ciliated cells among all the groups, which was consistent with the testicular immunoreactions (Figures 7A2–E2).

### Western Blotting Determination of T1R2, T1R3, and GNAT3 in Testis and Epididymis

**Figure 8:**
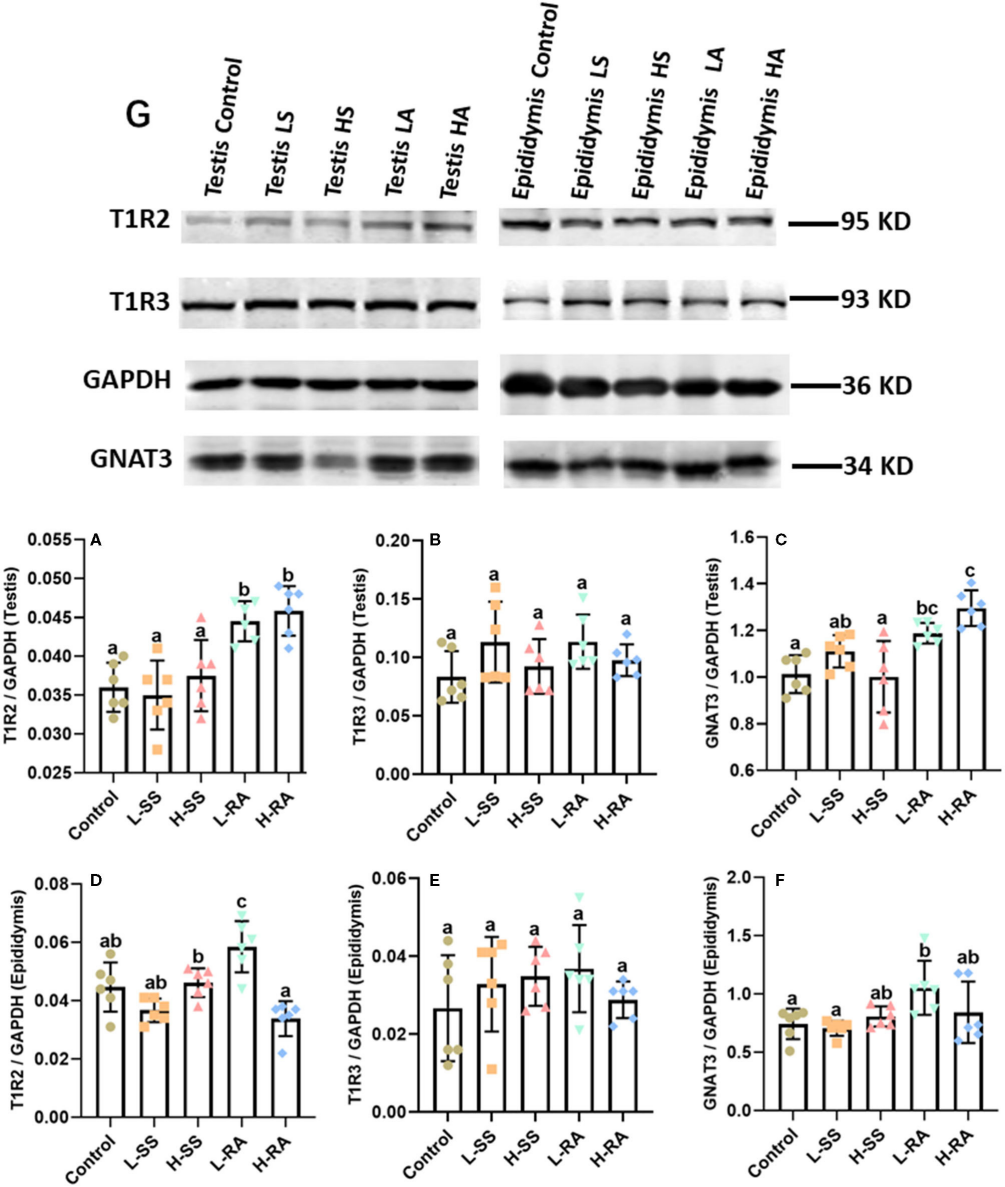
Effect of dietary non-nutritive sweetness drinking on the expression of sweet-tasting proteins T1R2, T1R3, and GNAT3 in testis and epididymis in male guinea pigs. Animals in the control, L-SS, H-SS, L-RA, and H-RA groups were supplied water solution with normal water, 1.5 mM sodium saccharin, 7.5 mM sodium saccharin, 0.5 mM rebaudioside A, and 2.5 mM rebaudioside A for 28 days, respectively. Equal amounts of protein lysate 50 μg were separated and incubated with primary antibodies of T1R2, T1R3, GNAT3, and GAPDH **(8G)**; GAPDH was used as an internal control. The intensities of blots were calculated with target protein bands to corresponding GAPDH **(8A–F)**. Data are shown as means ± SD (*n* = 6). Bars with different letters denote significant differences (*P* < 0.05).

We verified the visible immunoreaction changes with the Western blotting determination of the expression of T1R2, T1R3, and GNAT3 in the testis and epididymis (Figure 8). The testicular expression of T1R2 in L-RA and H-RA significantly increased compared with control, L-SS, and H-SS (Figure 8A, *P* < 0.05). Meanwhile, the expression of GNAT3 in L-RA and H-RA was significantly greater than that in control (Figure 8C, *P* < 0.05). Consistent with immunoreaction changes, there was no significant difference in the testicular epididymal expression of T1R3 among all the groups (Figures 8B,E, *P* > 0.05). Furthermore, the epididymal expression level of T1R2 and GNAT3 in L-RA was elevated significantly compared with control (Figures 8D,F, *P* < 0.05).

## Discussion

Saccharin sodium has been approved by the United States Food and Drug Administration (FDA) as a safe artificial sweetener. Consumption of non-nutritive sweetener is highly prevalent all over the world; well-controlled, prospective trails are required to understand the biological impact of this widespread NNS exposure (20). The reproductive consequences of non-nutritive sweetness in male are still unclear, which may be mediated by activation of sweet taste receptors in extraoral testis or epididymis. We selected two different types of NNS, chemical sodium saccharin and natural rebaudioside A, to study the influence on histologic change and expression of sweet taste receptors in testis and epididymis of young male guinea pigs.

A previous study on adult male mice showed that the body weight significantly increased in the 3.5 mM saccharin-treated group on weeks 4 and 5, occurring with significant cumulative differences in food intake and water consumption, while mice in the 7 mM saccharin group increased their water consumption significantly in the first 2 weeks (19). The results in male guinea pigs first showed that drinking a solution of 1.5 mM sodium saccharin significantly increased food intake but decreased water consumption on week 4; conversely, exposure to 7.5 mM sodium saccharin significantly reduced food intake but increased water consumption on week 4. However, rebaudioside A with a concentration of 0.5 mM and 2.5 mM both increased food intake on weeks 3 and 4 but had no influence on water consumption compared with control. The different administration dosage of sodium saccharin might result in different animal metabolic response (21). The mechanisms are likely synergistic and may differ across species [for example, genetic variants of sweet taste receptor gene associated with food intake (22) and chemically distinct non-nutritive sweeteners (14)]; therefore, we uncovered that dose-response metabolic manners to the equivalent sweetness NNS with artificial sodium saccharin and natural rebaudioside A were different in male guinea pigs.

The previous study revealed that saccharin sodium decreased male mice reproductive performance at a high dose (0.46 g/kg/day), while no adverse reproductive effects were observed at low (0.04 g/kg/day) and medium (0.21 g/kg/day) doses (18). The results showed that the growth performance in guinea pig testes and epididymis was impaired as a result of the different treatments of sodium saccharine and rebaudioside A. Consequently, the body weights of guinea pigs were associated with food intake. Significant food intake in L-SS and H-RA led to significant body weight compared with control. The results revealed that low-sweetness 1.5 mM sodium saccharin and high-sweetness 2.5 mM rebaudioside A had similar functional effects on food intake and body weight change in male guinea pigs, which were consistent with the previous findings that found the addition of sweetener to diets increased sweetness sensory-motivated intake (23) and then the induced greater body gain in male rats (24, 25). We speculated that NNS contributed to metabolic derangement (26, 27), such as increase in food intake and body weight gain (28). However, H-SS decreased food intake and had no significant change in body weight, and the prevailing thought of the authors was toxicological effects of high-intensity 7.5 mM sodium saccharin on food intake in male guinea pigs (29). A previous study found that there was no substantive change in testis weight from age of 56 days in rats (18). Parallel to the above results, there was no significant difference in relative testis weight in male guinea pigs with age of 56 days between the control group and the treated groups, in spite of greater body weight in L-SS and H-RA. It is mainly due to the fact that testis weight is stable and not closely associated with body weight at age of 56 days in rat and guinea pigs. Different from the relative testis weight, however, the mechanism of the unstable relative epididymis weight in male guinea pig with the age of 56 days needs to be further investigated.

Compared with control in this experiment, NNS contributes no significant influence on serum testosterone and estradiol level. Apart from testosterone, a lower circulating serum level of estradiol was detected in L-RA compared with L-SS. Meanwhile, water consumption on week 4 in L-RA was greater than that in L-SS, and body weight on week 4 in L-RA was significantly lesser than that in L-SS. The saccharin-exposure experiment in mice showed increased sweet taste receptor molecules and upregulated steroidogenic enzymes (19) and NNS tastant-evoked adenosine 3',5'-cyclic monophosphate (cAMP) signal (30), which was a key regulator of steroidogenesis in Leydig cells (31). However, whether serum estradiol level in male guinea pig relates to greater sweet solution consumption or heavier body weight is still rudimentary, since whether sweet taste receptors regulate steroidogenesis requires further investigation.

In this study, high-dose 7.5 mM sodium saccharin exerted adverse morphologic influences on testis and epididymis, markedly reduced number of Leydig cells, Sertoli cells, and germ cells in seminiferous tubules of the testis, and elongated and enlarged efferent ducts with a thinner layer of ciliated cells in the epididymis. We, herein, uncovered the positive biological functions of low-dose 0.5 mM rebaudioside A on morphologic changes, including testicular cohesive germ cells and an epididymal thicker layer of basal cells in epididymis ductus. Numerous studies suggested that high-intensity sodium saccharin contributed adverse biological, even toxicological, effects on reproductive systems (19, 32–35). Conversely, a certain intensity of rebaudioside A represented positive biological functions (36–38). A study showed that natural sweeteners exerted their sweet taste by specifically binding to sweet taste receptors (39). It was reported that the effects of NNS exposure on reproductive organ function were related to the expression of sweet taste receptor (19). This study showed that 0.5 mM rebaudioside A significantly elevated the testicular and epididymal expression of T1R2 and GNAT3 in male guinea pigs, including the stronger expression on germ cells and Sertoli cells in the testis, and basal cells and ciliated cells in the epididymis. A previous study has reported that genetic loss or pharmacological blockade of testes-expressed taste genes causes male sterility (17). Hence, we estimated that low-dose rebaudioside A enhanced testicular and epididymal functions by activating the expression of T1R2 and GNAT3. The functions of sweet taste receptors in spermatozoa are related to cAMP concentration (16), which are essential to produce steroid hormone associated with testicular and epididymal functions. The current increase in the testicular expression of T1R2 and GNAT3 in H-RA indicated that high-intensity rebaudioside A still represented positive effects on testicular functions, consistent with the conclusion that rebaudioside A causes no acute and subacute toxicity (40), even at 2.5% dietary concentration (29).

The testicular and epididymal expression of T1R3 in this study had no significant changes among all the groups, and this observation suggested that the testicular and epididymal function in male guinea pigs exposed to NNS was maintained by the Gα-mediated pathway without T1R3 (18) and that the effect of the agonist was to pull the bottom part of VFD3/T1R3 toward the bottom part of VFD2/T1R2 (41). Male guinea pigs exposed to 1.5 and 7.5 mM sodium saccharin represented no effect on the expression of sweet taste receptors, such as T1R2, T1R3, and GNAT3. According to the adverse histologic changes in testis and epididymis in H-SS, one explanation for this was that sodium saccharin at certain concentration turned to bind bitter taste receptors instead of sweet taste receptors (42, 43), since NNS causes aftertaste bitterness in addition to sweetness (44). Therefore, whether NNS has influences on the expression of bitter taste receptors requires further study on young guinea pigs from prepubertal to peripubertal age.

## Conclusions

The data clearly suggest that saccharin sodium with high-dose sweetness has potential adverse biologic effects on testicular development and that rebaudioside A functions as a potential steroidogenic disruptor in male guinea pigs. Based on the study results, we recommend that additional systemic reproductive studies be performed to verify the safety of a high dose of non-nutritive sweeteners on the male reproductive tract.

## Data Availability Statement

The original contributions generated for the study are included in the article/supplementary material, further inquiries can be directed to the corresponding author/s.

## Ethics Statement

Animals were housed in Zhejiang Chinese Medical University Laboratory Animal Research Center with experiment facility license of SYXK(ZHE)2018-0012, animal experiment process conforms to the principle of the animal protection, the animal welfare and the ethics as well as the related stipulation on National Experimental Animal Welfare Ethics (China) with approval number of IACUC-20181224-13.

## Author Contributions

JL designed, drafted, and contributed to the experiment design and study corrections. TS carried out the experiment and analyzed the data. All authors contributed to the article and approved the submitted version.

## Conflict of Interest

The authors declare that the research was conducted in the absence of any commercial or financial relationships that could be construed as a potential conflict of interest.

## Publisher's Note

All claims expressed in this article are solely those of the authors and do not necessarily represent those of their affiliated organizations, or those of the publisher, the editors and the reviewers. Any product that may be evaluated in this article, or claim that may be made by its manufacturer, is not guaranteed or endorsed by the publisher.
